# Count every newborn; a measurement improvement roadmap for coverage data

**DOI:** 10.1186/1471-2393-15-S2-S8

**Published:** 2015-09-11

**Authors:** Sarah G Moxon, Harriet Ruysen, Kate J Kerber, Agbessi Amouzou, Suzanne Fournier, John Grove, Allisyn C Moran, Lara ME Vaz, Hannah Blencowe, Niall Conroy, A Metin Gülmezoglu, Joshua P Vogel, Barbara Rawlins, Rubayet Sayed, Kathleen Hill, Donna Vivio, Shamim A Qazi, Deborah Sitrin, Anna C Seale, Steve Wall, Troy Jacobs, Juan Gabriel Ruiz Peláez, Tanya Guenther, Patricia S Coffey, Penny Dawson, Tanya Marchant, Peter Waiswa, Ashok Deorari, Christabel Enweronu-Laryea, Shams El Arifeen, Anne CC Lee, Matthews Mathai, Joy E Lawn

**Affiliations:** 1 Maternal, Adolescent, Reproductive and Child Health (MARCH) Centre, London School of Hygiene and Tropical Medicine, London, WC1E 7HT, UK; 2 Department of Infectious Disease Epidemiology, London School of Hygiene and Tropical Medicine, London, WC1E 7HT, UK; 3 Saving Newborn Lives, Save the Children, 2000 L Street NW, Suite 500, Washington, DC 20036, USA; 4 Data, Research and Policy, UNICEF, 3 UN Plaza, New York City, NY 10017, USA; 5 Children's Investment Fund Foundation (CIFF), Health Team, 7 Clifford Street, London, W1S 2FT, UK; 6 Maternal, Newborn and Child Health, Global Development Program, Bill & Melinda Gates Foundation, Seattle, WA 98109, USA; 7 Office of Health, Infectious Disease and Nutrition, Bureau for Global Health, United States Agency for International Development, Washington, DC 20523-1000, USA; 8 Mohamed Aden Sheikh Children's Teaching Hospital, Hargeisa, Somaliland; 9 UNDP/UNFPA/UNICEF/WHO/World Bank Special Programme of Research, Development and Research Training in Human Reproduction (HRP), Department of Reproductive Health and Research, World Health Organization, Avenue Appia 20, CH-1211, Geneva, Switzerland; 10 Maternal and Child Survival Program/Jhpiego, 1776 Massachusetts Avenue, Suite 300, Washington, DC 20036, USA; 11 Saving Newborn Lives, Save the Children in Bangladesh, House 35, Road 43, Gulshan 2, Dhaka 1212, Bangladesh; 12 USAID Applying Science to Strengthen and Improve Health Systems (ASSIST) project, University Research Co. LLC (URC), 7200 Wisconsin Avenue, Suite # 500 Bethesda MD 20814, USA; 13 United States Agency for International Development, Bureau for Global Health, Office of Health, Infectious Disease and Nutrition, Maternal and Child Health Division, 1300 Pennsylvania Avenue, NW, Washington, DC 20523, USA; 14 Department of Maternal, Newborn, Child and Adolescent Health, World Health Organization, 20 Avenue Appia, 1211 Geneva 27, Switzerland; 15 University College London Department of Infectious Diseases Informatics, UCL Institute for Health Informatics, Farr Institute, 222 Euston Road, London NW1 2DA, UK; 16 School of Medicine, Pontificia Universidad Javeriana, Carrera 7 No 40-62, Bogotá, Colombia; 17 Fundación Canguro, Calle 56A No 50-36 - Bloque A13, Apto 416, Pablo VI Azul, Bogotá, Colombia; 18 Hospital Universitario San Ignacio, Carrera 7 No 40-62, Bogotá, Colombia; 19 Devices/Tools Global Program, Health Technologies for Women and Children, PATH, 2201 Westlake Avenue, Suite 200, Seattle, 98121, USA; 20 International Division, John Snow Inc. (JSI), 44 Farnsworth St., Boston, MA 02210-1211, USA; 21 Department of Disease Control, London School of Hygiene and Tropical Medicine, London, WC1E 7HT, UK; 22 Maternal and Newborn Working Group, INDEPTH Network, 38 & 40 Mensah Wood Street, East Legon, P.O. Box KD213 Kanda, Accra, Ghana; 23 Makerere University College of Health Sciences, School of Public Health, Plot 1 New Mulago Hospital, P.O Box 25809, Kampala, Uganda; 24 Department of Paediatrics, WHO Collaborating Centre for Education & Research in Newborn Care, All India Institute of Medical Sciences, Ansari Nagar, New Delhi, 110029, India; 25 Department of Child Health, School of Medicine and Dentistry, College of Health Sciences, University of Ghana, Accra, PO Box 4236, Ghana; 26 Centre for Child and Adolescent Health, International Centre for Diarrhoeal Disease Research, Bangladesh (ICDDR,B) 68, Shaheed Tajuddin Sharani, Mohakhali, Dhaka 1212, Bangladesh; 27 Department of Pediatric Newborn Medicine, Brigham and Women's Hospital, 75 Francis Street, Boston, MA 02115, USA

**Keywords:** Newborn, maternal, coverage, indicators, monitoring, evaluation, quality, equity, impact, mortality, stillbirth, health systems, accountability

## Abstract

**Background:**

The *Every Newborn *Action Plan (ENAP), launched in 2014, aims to end preventable newborn deaths and stillbirths, with national targets of ≤12 neonatal deaths per 1000 live births and ≤12 stillbirths per 1000 total births by 2030. This requires ambitious improvement of the data on care at birth and of small and sick newborns, particularly to track coverage, quality and equity.

**Methods:**

In a multistage process, a matrix of 70 indicators were assessed by the *Every Newborn *steering group. Indicators were graded based on their availability and importance to ENAP, resulting in 10 core and 10 additional indicators. A consultation process was undertaken to assess the status of each ENAP core indicator definition, data availability and measurement feasibility. Coverage indicators for the specific ENAP treatment interventions were assigned task teams and given priority as they were identified as requiring the most technical work. Consultations were held throughout.

**Results:**

*ENAP *published 10 core indicators plus 10 additional indicators. Three core impact indicators (neonatal mortality rate, maternal mortality ratio, stillbirth rate) are well defined, with future efforts needed to focus on improving data quantity and quality. Three core indicators on coverage of care for all mothers and newborns (intrapartum/skilled birth attendance, early postnatal care, essential newborn care) have defined contact points, but gaps exist in measuring content and quality of the interventions. Four core (antenatal corticosteroids, neonatal resuscitation, treatment of serious neonatal infections, kangaroo mother care) and one additional coverage indicator for newborns at risk or with complications (chlorhexidine cord cleansing) lack indicator definitions or data, especially for denominators (population in need). To address these gaps, feasible coverage indicator definitions are presented for validity testing. Measurable process indicators to help monitor health service readiness are also presented. A major measurement gap exists to monitor care of small and sick babies, yet signal functions could be tracked similarly to emergency obstetric care.

**Conclusions:**

The ENAP Measurement Improvement Roadmap (2015-2020) outlines tools to be developed (e.g., improved birth and death registration, audit, and minimum perinatal dataset) and actions to test, validate and institutionalise proposed coverage indicators. The roadmap presents a unique opportunity to strengthen routine health information systems, crosslinking these data with civil registration and vital statistics and population-based surveys. Real measurement change requires intentional transfer of leadership to countries with the greatest disease burden and will be achieved by working with centres of excellence and existing networks.

## Background

The close of the Millennium Development Goals (MDGs), with a halving of maternal mortality and under five child deaths, demonstrates that global targets are linked to national and global accountability and can drive change. Under-five deaths due to HIV/AIDS, malaria and measles (among others), have seen the greatest proportional declines [1]. Where indicators for high impact, evidence-based interventions are carefully tracked, previous analysis has demonstrated that coverage tends to improve, largely due to focused policy attention, investment and informed planning, leading to better population health outcomes [2]. Interventions for child health and causes of child death have had more programmatic data (coverage and process), collected more frequently, at a more granular level (e.g. district level, by various equity analyses groups), than for newborn health, where the data is of poorer quantity and quality, and has been collected with less frequency [3].

As the MDGs transition to the Sustainable Development Goals (SDGs), there remains an unfinished agenda for 2.7 million neonatal deaths, for whom progress has been much slower than progress towards reducing the overall under 5 mortality rate. An estimated 2.6 million stillbirths were not counted at all in the MDGs[4]. Well-functioning civil registration and vital statistics (CRVS) systems generate policy, ensure access to services and are associated with better health outcomes worldwide [5]; counting births and deaths, especially the deaths around the time of birth, lies at the heart of post-2015 health monitoring, accountability and action [3]. Tracking vital events and measuring coverage is also central to developing national health management information systems (HMIS), such as in the Measurement and Accountability for Results in Health (MA4Health) Roadmap [6], which aims to increase investment in national data systems and data use.

The *Every Newborn *Action Plan (ENAP) [7] is a global multi-partner movement to end preventable maternal and newborn deaths and stillbirths. Through a series of consultations, multiple stakeholders (governments, United Nations (UN) agencies, donors, business communities, professional associations, academic and research institutions, global initiatives and civil society members) developed an impact framework and an action and measurement agenda for integration within national newborn health plans [3,8].

**Figure 1:**
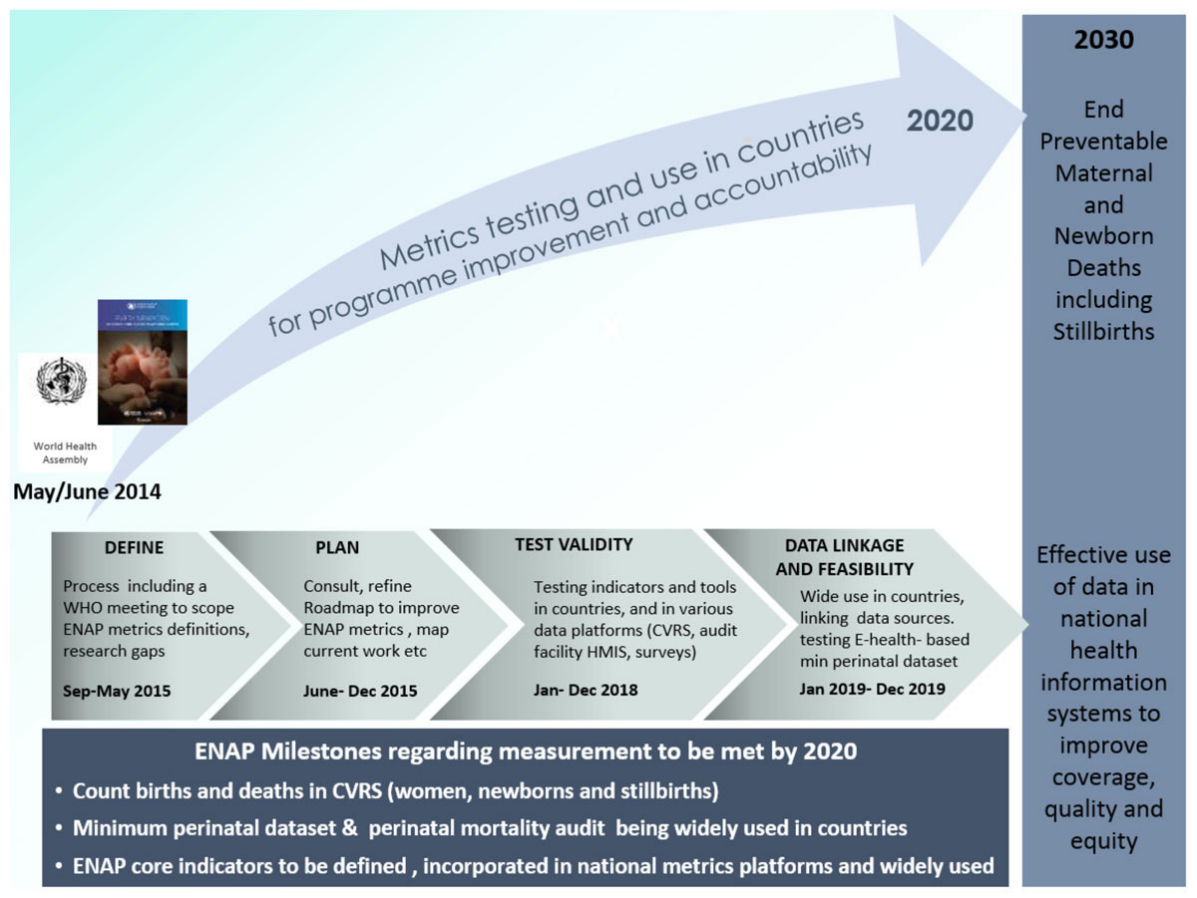
***Every Newborn *Action Plan (ENAP) Measurement Improvement Roadmap: the arc of change**. In support of Health Measurement and Accountability post-2015: A Common Roadmap WHO (2015) [6]. ENAP: Every Newborn Action Plan; WHO: World Health Organization.

To reach 2030 national targets for neonatal mortality and stillbirth rates of ≤12 per 1000 births, high and equitable coverage of the evidence-based interventions identified by ENAP is needed [9]. ENAP prioritises achieving universal coverage of these interventions particularly during childbirth and the first week of life. Yet many of these interventions are not systematically measured. One of the five ENAP strategic objectives - to count every newborn (and birth) - underlines the need for improved data and accountability. The ENAP milestones, linked to a World Health Assembly resolution [7], have a particular focus on inputs required prior to 2020 and more than half refer to improving metrics for targeting and driving change (Figure 1). One such milestone is to develop a monitoring framework building on the Commission on Information and Accountability (COIA) for Women's and Children's Health [10] to track global progress post 2015 and align with country priorities and objectives.

The principal focus of this paper is based on the ENAP milestone to define and improve priority coverage indicators, as this was where the largest measurement gaps were identified. Many newborn care interventions lack standard indicator definitions and are not routinely monitored at national or global level, especially in low and middle-income countries (LMIC). We define a coverage indicator as a population-level metric that measures the number of individuals that receive an intervention or service (numerator) out of a total population that should receive the intervention or service (the denominator). For the numerator, indicators rely on clear technical definitions of the service or intervention. Where there is difficulty capturing the population in need (the denominator) particularly for specific treatment interventions, some indicators (such as the caesarean-section rate) use total live births as the denominator to give a proxy. In such cases, where the aim is not for 100% coverage, the rate is then benchmarked against a target threshold.

The coverage indicators prioritised by the Commission for Information and Accountability (COIA) mainly reflect contact points along the continuum of care, notably antenatal care, skilled birth attendance and postnatal care. Such coverage indicators capture contact with the health system or delivery of a specific intervention, but not always detailed, accurate information on the content or quality of the care delivered [11], although antenatal care now has a detailed content module within the Demographic and Health Survey (DHS) [12]. In high-burden countries the main current data source is through household surveys. The most commonly employed household surveys are the United States Agency for International Development (USAID)-supported DHS [13] and the United Nations International Children's Fund (UNICEF)-supported Multiple Indicator Cluster Survey (MICS) [14]. However, coverage of many maternal and newborn interventions cannot feasibly and/or accurately be collected through household surveys.

For health information collected through household surveys, the data quality usually depends on the validity of the mother's report, often up to two to five years after the intervention occurred. There is evidence suggesting that maternal recall of events that occurred during labour is poor [12], especially if there were complications. In addition, how the question is asked can affect the accuracy of the response. For surveys, large sample sizes are needed to generate sufficient statistical power to assess social and demographic factors. Bryce et al: [15] described some of the limitations of household surveys for measuring coverage of interventions, including the time, cost and limited validity (sensitivity and specificity) of many of the indicators.

Health facility assessments (HFAs) are frequently used to complement HMIS, facility-based logistics and service delivery information systems. These provide information on staffing, equipment availability, spatial organisation, data collection capacity, and service readiness. A number of standardised HFA tools exist, the most commonly employed being the Service Availability and Readiness Assessment (SARA) [16], Service Provision Assessments (SPA) [17] and the Emergency Obstetric Care (EmOC) needs assessments [18]. These allow health systems to report on a sample of facilities that provide a certain service or have health workers trained in specific skills, but are not routine reporting mechanisms. In addition, the WHO Health Access/Action International database has data on medication availability. Service availability and quality indicators provide complementary metrics to population coverage which can be used to ensure that services achieve adequate coverage and give due attention to the availability of care, and the readiness of facilities to deliver the safe and quality care that is fundamental to the *Every Newborn *movement.

Since coverage of evidence-based care for mothers and newborns is often unknown, or data may be old or not locally available, this is a major "bottleneck", impeding scale up of high-impact, evidence-based interventions for newborns. Such data have been critical in accelerating progress in the implementation and scale-up of immunisation and HIV programmes through increased policy attention, focused investment of resources and better accountability structures [15]. Such data are critical for informed planning, driving programme improvement and targeting underserved populations to reduce inequities.

The objectives of this paper are to:

1. Describe the systematic process used to select ENAP indicators and present the core and additional indicators.

2. Assess the status (technical definitions and data availability) of the ENAP coverage indicators and identify actions needed to improve these for measurement at scale, particularly for coverage of the treatment interventions.

3. Identify priorities for testing validity and feasibility, in order to institutionalise these metrics within large scale data collection platforms and outline a five-year measurement improvement roadmap.

## Methods

### Objective 1: systematically grade to select the ENAP core and additional indicators

A multi-stage process was carried out to identify a list of potential indicators and then prioritise a short list. This process involved a working group appointed by the ENAP management team who compiled a comprehensive list of indicators, drawing on existing databases such as COIA [10], UNICEF's State of the World's Children (SoWC) [19], Countdown to 2015 [20] and other World Health Organization (WHO) statistics and reports. Standardised, nationally representative survey tools currently in use (MICS, DHS, SPA, SARA and EmOC surveys) were considered as sources of data. In addition, possible indicators relating to common causes of neonatal death were included. This resulted in a matrix of over 70 relevant indicators measuring impact (mortality and morbidity), outcome (coverage of care for all babies and coverage of treatment interventions), outputs (service quality, availability, demand, and the enabling environment) and inputs (human resources, essential medicines and supplies) (see Additional file 1). The current status of definitions, measurability and data availability were reviewed for each of the proposed indicators.

A systematic scoring process was applied to prioritise core indicators that could track the main focus of the action plan, particularly on quality of care at birth and the five strategic objectives. Each indicator was graded by its importance to the ENAP focus (A to C) and by current data availability (1 to 3). A grade of A was given to indicators of highest relevance and match to the ENAP focus and a score of 1 was given to indicators with a common and consistent definition already measured in existing data sources. Scoring was completed by an expert working group and decided via group consensus with priority given to indicators in terms of their relevance to the ENAP focus, rather than data availability.

Given the principle of accelerating impact, a decision was taken to focus on a shorter list of important indicators and ensure those would be made measureable, rather than to just select those that were already measureable. Hence, indicators were prioritised first based on their importance to the ENAP focus (category A) and then on data availability. Indicators in Category A ranged from those with definitions and existing data (availability 1) to those without standard definitions and existing data (availability 2 or 3). The latter were identified as having priority measurement gaps that needed to be addressed with a specific program of work.

### Objective 2: assess status of ENAP coverage indicators and identify priorities to improve measurement at scale

**Figure 2:**
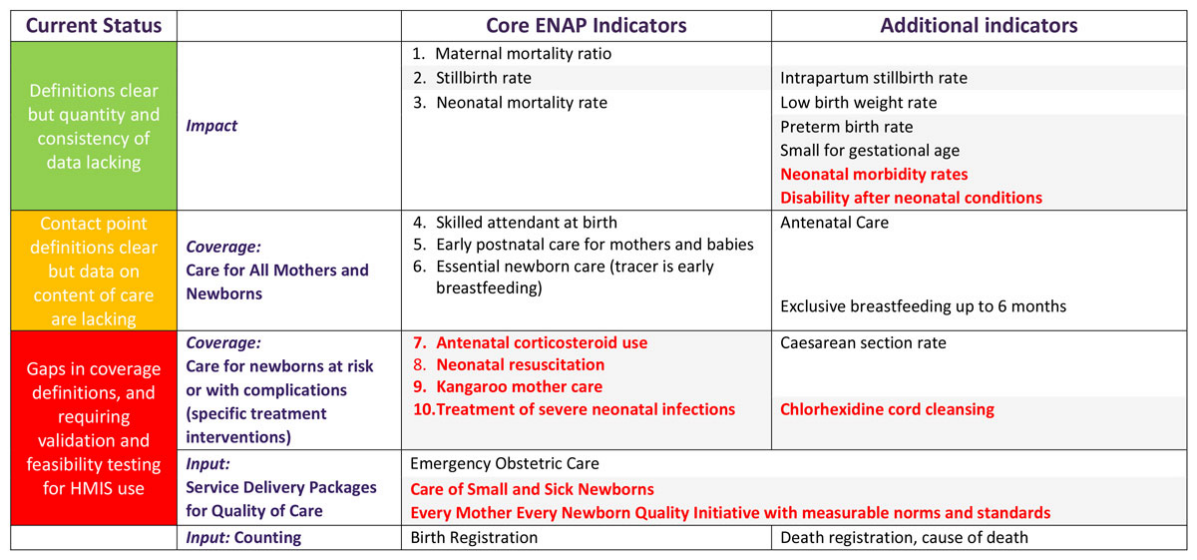
***Every Newborn *Action Plan (ENAP) core and additional indicators**. Shaded= Not currently routinely tracked at global level. Bold red= Indicator requiring additional testing to inform consistent measurement. Indicators to be disaggregated by equity such as urban/rural, income and education. Adapted from WHO and UNICEF, Every Newborn Action Plan. WHO, 2014. http://www.everynewborn.org/ and Mason *et al*: Lancet 2014.

For each of five high impact interventions identified with the greatest measurement gap (red box in Figure 2), a Task Team was established. These included antenatal corticosteroids (ACS), newborn resuscitation, Kangaroo Mother Care (KMC), case management of serious neonatal infection and chlorhexidine cord cleansing. The Task Teams sought to represent both the maternal and newborn health communities and reflect multiple stakeholders, e.g. non-governmental organisations, UN organisations, professional associations, and research institutions; ensuring representation from LMIC. With the support of the ENAP metrics coordination group, Task Teams carried out a consultation process to define indicators based on a technical definition, suggest feasible indicators that can be measured now through existing data collection platforms, and outline research priorities to test validity and feasibility for these coverage metrics for each area, including data collection tools.

WHO hosted a consultation at a meeting in Geneva, December 2014 to review the work of the Task Teams, and also gain inputs on the other core indicators. This meeting developed a draft plan to deliver on the ENAP metrics milestones, including discussion on the specific actions needed to improve coverage indicators. Plans for improving measurement tools and tracking systems were also discussed; for example, perinatal audit tools, neonatal care registers and Civil Registration and Vital Statistic (CRVS) improvements. The draft plan was then advanced by those at the meeting and through wider consultation.

The priorities for testing validity and feasibility to institutionalise these metrics within large scale data collection platforms and the measurement improvement roadmap (***Objective 3***) are discussed in detail in the discussion section of this paper.

## Results

### Objective 1: systematically grade to select the ENAP core and additional indicators

**Figure 3:**
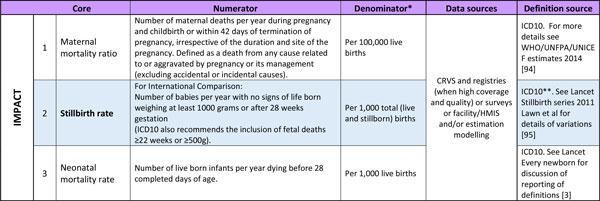
***Every Newborn *Action Plan (ENAP) core indicators regarding impact, with definitions and data sources**. Shaded= Not currently routinely tracked at global level. Bold= indicator requiring additional evaluation for consistent measurement. *The time period will normally be calculated per year. **ICD assumes weight and gestational age are equivalent, which they are not (see Stillbirth series Lawn *et al*: 2011). ICD: International Classification of Disease; UNFPA: United Nations Population Fund; UNICEF: United Nations International Children's Emergency Fund; WHO: World Health Organization.

Following the process described above, ENAP listed 10 core indicators (Figure 2). For the three impact indicators that already have agreed definitions (Figure 3), the priority is for improved quality and quantity of data. There is increasing consensus on the need to invest in CRVS and linked facility-based tracking to improve reliability of impact indicators [3,4,21].

**Figure 4:**
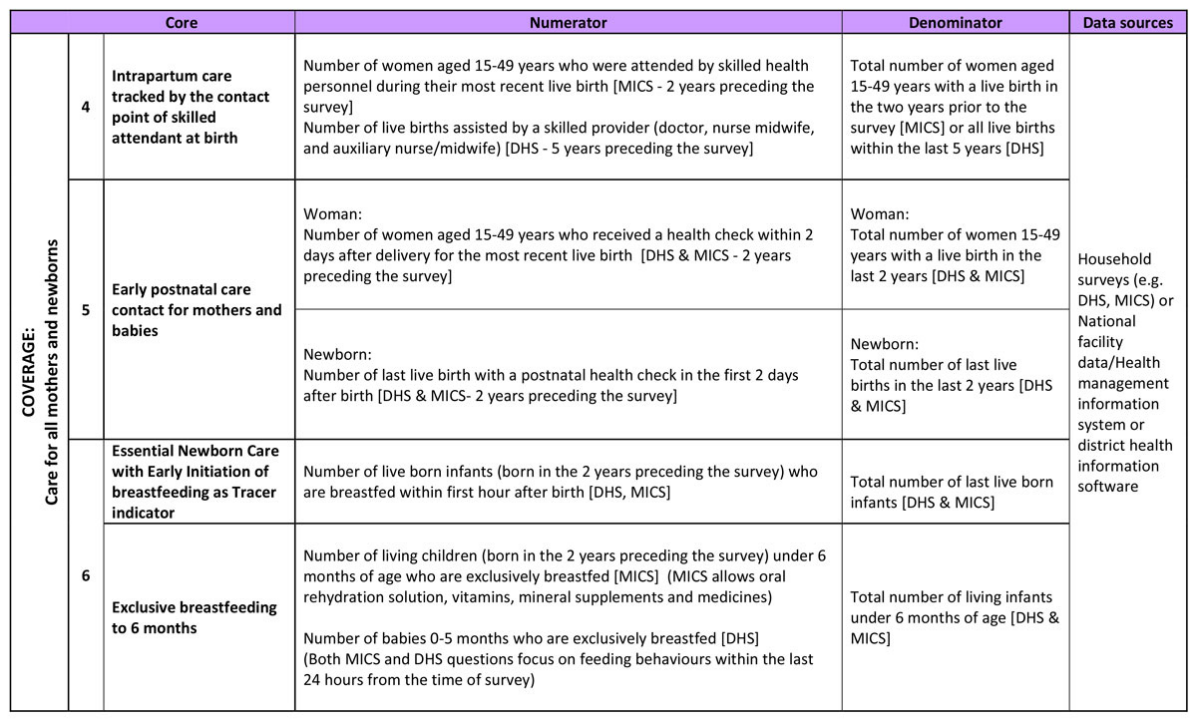
***Every Newborn *Action Plan (ENAP) core indicators regarding coverage of care for all mothers and newborns, with definitions and data sources**. DHS: Demographic and Health Survey; MICS: Multiple Indicator Cluster Survey.

**Figure 5:**
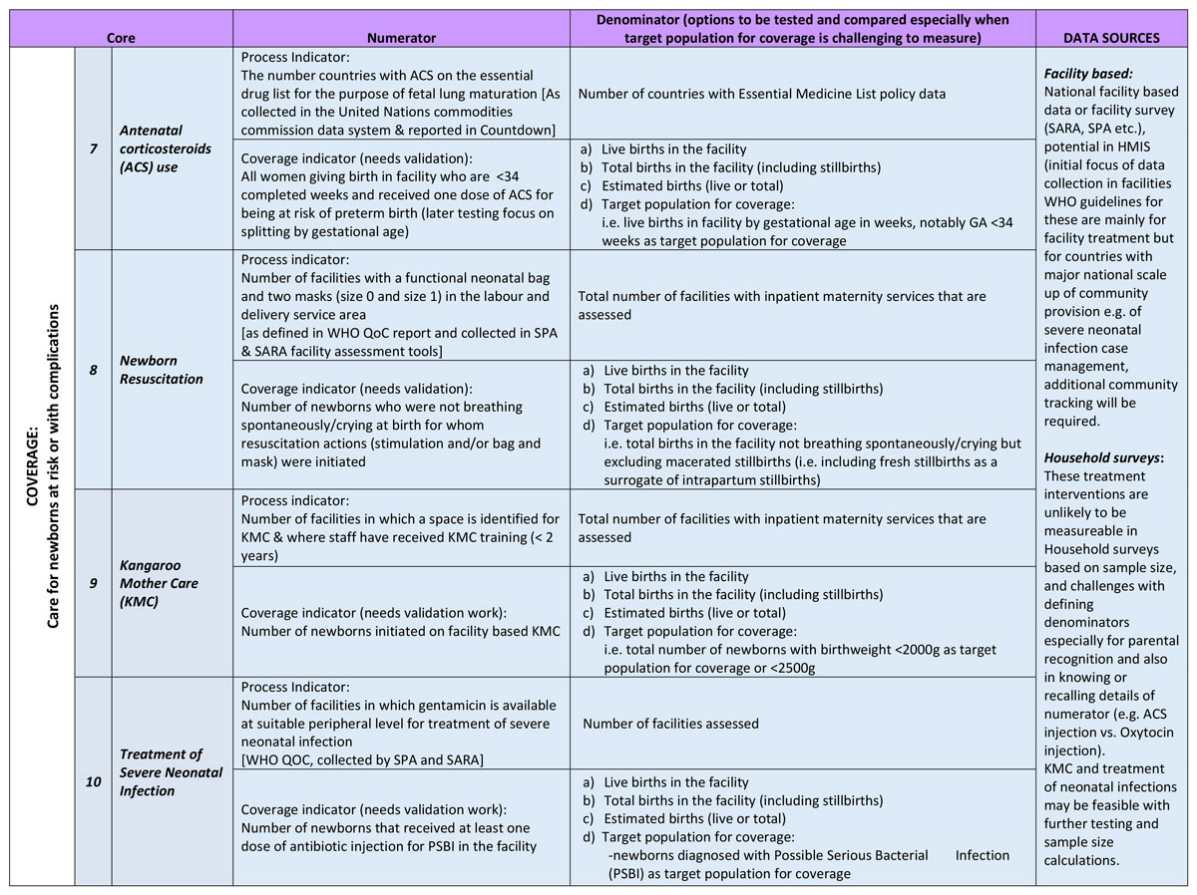
**Every Newborn Action Plan (ENAP) core indicators regarding coverage of care for newborns at risk or with complications, with definitions and data sources**. Blue coloured cells= not currently tracked and collated by United Nations. Bold italics= indicator needing further work to ensure availability of consistent data in routine information systems. All coverage indicators to be tracked in such a way that they can be broken down to assess equity- e.g. urban or rural, regional, wealth quintile. ACS: antenatal corticosteroids; GA: gestational age; HMIS: Health Management Information System; KMC: kangaroo mother care; QoC: quality of care; SARA: Service Availability and Readiness Assessments; SPA: Service Provision Assessments; WHO: World Health Organization.

**Figure 6:**
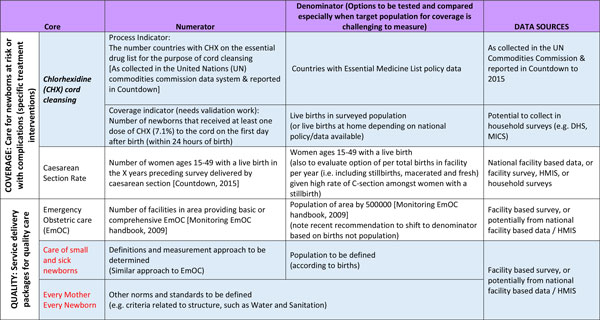
***Every Newborn *Action Plan (ENAP) core indicators regarding coverage of complications and extra care (specific treatment indicators), with definitions and data sources**. Blue coloured cells= not currently tracked and collated by United Nations. Bold italics= indicator needing further work to ensure availability of consistent data in routine information systems. Red= service delivery package for which norms and standards will be defined and tracked. All coverage indicators to be tracked in such a way that they can be broken down to assess equity- e.g. urban or rural, regional, wealth quintile. CHX: chlorhexidine; DHS: Demographic and Health survey; EmOC: emergency Obstetric Care; HMIS: Health Management Information System; MICS: Multiple Indicator Cluster Survey; UN: United Nations.

The principal focus of this paper is on the coverage indicators, where the largest metrics gaps were identified. The coverage indicators fall into two groups: key contact points for care for all mothers and newborns (Figure 4), and specific treatment interventions (mainly for care for newborns at risk or with complications) (Figure 5 and 6). For essential newborn care, early initiation of breastfeeding was identified as a tracer indicator, with exclusive breastfeeding up to 6 months as an additional indicator. Chlorhexidine cord cleansing was also added to the improvement agenda, given the gaps in coverage data.

### Objective 2: assess status of ENAP coverage indicators, and identify priorities to improve measurement at scale

For each coverage indicator, we describe technical definitions, current data availability, improvements needed and steps to be taken.

## Coverage: care of all mothers and newborns (contact points)

### Intrapartum care

#### Technical definition of package

A package of support and healthcare around the time of birth integral to maintaining perinatal and maternal safety along the continuum of care [9,22]. Skilled birth attendance is used as the contact point indicator to monitor coverage of this care.

#### Indicator to track contact point

A skilled birth attendant (SBA) is described by the WHO as an accredited health professional (such as a midwife, doctor or nurse) educated and trained to proficiency in the skills needed to manage normal (uncomplicated) pregnancies, childbirth and the immediate postnatal period and in the identification, management and referral of complications in women and newborns [23].

#### Current data availability

SBA data are available mostly from DHS, MICS, and are reported in many UN documents and by the Countdown to 2015 report series, which charts country progress towards meeting MDG goals and targets. However, no robust time series has been published for all countries for the MDG era to date, although SBA was the main indicator under MDG5 for maternal health. Of 75 countries participating in Countdown, all but 15 provide equity analysis in relation to the coverage of SBA [20] (countries who do not report equity compared with SBA coverage are: Angola, Botswana, Brazil, China, Djibouti, Equatorial Guinea, Eritrea, Korea, Mexico, Myanmar, Papua New Guinea, Solomon Islands, South Sudan, Sudan and Turkmenistan). These suggest SBA coverage has the widest equity gap for any contact point along the COIA continuum of care indicators [20]. SPA also has a new optional observational module for labour and delivery care that has been applied in Kenya, Malawi and Bangladesh developed by the Maternal and Child Health Integrated Program (MCHIP) that provides supplementary data for assessment of quality of care.

#### What can we do to improve the data?

While WHO's definition of a SBA has a defined list of core midwifery skills [18], measurement of SBA is challenged by the variety of cadres included in the definition and the lack of consistency in training, skills and core functions across countries [24]. Besides doctors, nurses and midwifes, there are several other country specific cadres of auxiliary midwifes, medical assistants and other health professionals that are included in the SBA category in many countries; these may also be subject to change over time, or across survey programmes. Current work towards standardising the professional remit of SBAs and foster more universal accountability mechanisms are being carried out by WHO, UNICEF and UNFPA. Expert consultations will be held in late 2015 to discuss operational definitions and develop measurement guidance for survey programmes.

In addition, SBA is an indicator of contact with the health system and does not provide information on the content or quality of care making it an incomplete and misleading proxy for quality of care at birth [12]; additional information about equipment, provider skills, referral availability, content of care and other measures of quality are also required. Process indicators on facility readiness are collected by SPA, SARA and EmOC needs assessments (Figure 4) though the range of data collected varies between surveys and there is limited focus on newborn care. Current DHS and MICS survey tools do not collect extensive data on the content of care at time of birth [12]; therefore, increasing the capacity and availability of routine facility level data is a priority for improvement.

### Early postnatal care

#### Technical definition of package

A package of healthcare provided to women and their newborn either at the facility or during consultation at home. For women who deliver at a health facility, WHO recommendations support inpatient care for at least 24 hours, and/or provision of care as early as possible and at least within 24 hours for women and newborns who are born at home [25].

#### Indicator to track contact point

Early postnatal care is defined as a contact provided to a woman and her newborn during the 2 days (48 hours) following birth (whether in a facility or at home) (see Figure 4) and excludes immediate postpartum care [13].

#### Current data availability

The early postnatal care contact point is measured in household surveys as two separate indicators (a postnatal health check for the newborn and a postnatal health check for the mother) tracking coverage of a first postnatal contact within 2 days of delivery. The questions used to derive this indicator have changed significantly over time and have been different between the DHS and MICS [14], however Phase 7 of DHS [13] now includes questions allowing computation of a comparable postnatal care indicator.

#### What can we do to improve the data?

Postnatal care is a package of services for women and babies, therefore, data on content and quality are required in addition to tracking the contact point. One critical question is to ensure the data can distinguish between intrapartum and postnatal care [26]. In both DHS and MICS, this is being attempted through the use of question prompts to better describe the content of the postnatal check and recent revision of the DHS core questionnaire includes a question on the content of PNC checks. Supplementary data pertaining to the content of care, provider skill and other quality control measures is urgently required; a move away from intermittent survey based data collection towards sustainable HMIS is essential in ensuring that effective management mechanisms can be facilitated and support routine quality of care tracking.

### Essential newborn care

#### Technical definition of package

Preventive and supportive care required for all newborns including: warmth, cleanliness, breastfeeding, cord and eye-care, Vitamin K and immunisations [27-29].

#### Indicator to track care

Due to the strong evidence of a reduction in newborn mortality and morbidity with early initiation of breastfeeding, especially through decreased rates of infection [30-32], early initiation of breastfeeding was prioritised as a tracer indicator for essential newborn care, with exclusive breastfeeding at 6 months as a further marker (Figure 4). Indicators of other components such as skin-to-skin care, may also be possible, and are recalled accurately by mothers [12]. However, these data are not currently widely available, and further testing is required to ensure that routine skin-to-skin can be accurately distinguished from KMC by survey respondents.

The WHO recommends the early initiation of breastfeeding within one hour of birth [33] and then exclusive breastfeeding for the first 6 months of life [34]. To support this, babies should be placed skin to skin with their mothers immediately following birth and offered help to breastfeed when needed [35].

#### Current data availability

MICS, DHS and other national household surveys collect data measuring coverage of the early initiation of breastfeeding [1,36] and it is reported in Countdown and SoWC [20]. Both MICS and DHS contain measurement questions focusing on feeding behaviours within the last 24 hours from the time of survey. This approach allows for more accurate recall of the behaviour, however, does not capture breastfeeding practises across the infant time period and, therefore, the results may not reflect breastfeeding practises over time.

#### What can we do to improve the data?

A recent validation study reported that the early initiation of breastfeeding indicator had high sensitivity (0.82) but poor specificity (0.25), using a household survey instrument [12]. Although the instrument used in the study posed a slightly different question than what is in DHS, this suggests a need for further testing and validation. Additional research to determine the impact of other essential newborn care practices would enable more informed and targeted behaviour change and associated measurement approaches.

## Coverage: care for newborns at risk or with complications (specific treatment interventions)

### Antenatal corticosteroids

#### Technical definition of intervention

Currently, antenatal corticosteroid therapy (ACS) (24 mg of intramuscular dexamethasone or betamethasone in divided doses over 24 hours) is recommended by WHO for all mothers at risk of imminent preterm birth (delivery before 34 completed weeks of gestation) when the mother is in a facility where accurate gestational age can be obtained, where there is no clinical evidence of maternal infection, and there are adequate levels of maternity care and special newborn care available [37] (WHO guidelines are currently being revised). These guidelines reflect changes after the Antenatal Corticosteroids Trial (ACT) which evaluated prescription of ACS at lower levels of the health system, with approximately half of births occurring at home, and found a risk of adverse outcomes especially amongst births after 34 completed weeks of gestation [38]. This trial underlines the importance of measuring gestational age, and better tracking of coverage and outcomes.

#### Current coverage data availability

Coverage data on provision of ACS for neonatal admissions are routinely collected within most high income countries (HIC), but are not consistently part of HMIS or standardised facility surveys. Since the intervention is used in health facilities [38], improved facility level data are a priority for capturing ACS coverage. Household surveys are unlikely to be a useful source for this information, as mothers may not accurately report ACS (with difficulties to differentiate between ACS and other drugs given at the time of labour). In addition, data may have low statistical power given the relatively small numbers in the population who receive ACS for fetal lung maturation [12].

#### Process indicator to track now

In many LMICs, where HMIS does not capture ACS coverage, a commodities-based process indicator can be measured for tracking in the short term; SARA and SPA includes the availability of dexamethasone within their facility checklist. WHO Health Access/Action International database also collect data on availability of dexamethasone and betamethasone in their existing pharmacy and facility audits [39]. However, a denominator of all health facilities may not be fully accurate as not all facilities would meet WHO criteria for safe provision of ACS (see definition above), including provision of appropriate maternal and newborn care [1,40]. Countdown reports the number of countries whose national policy recommends antenatal corticosteroids for preterm labour [1]. While this indicator is distal to coverage, it is available and helpful in tracking changes in policy context (Figure 5).

#### What can we do to improve the data?

It is challenging to define a precise indicator that can capture both eligible women who should receive ACS and measure ACS provision. Recent evidence suggests use of ACS may be associated with a risk of adverse outcomes for babies whose gestational age is ≥34 completed weeks [38]. A major challenge is defining the denominator of eligible mothers presenting in labour <34 weeks. In LMICs, the recall of last menstrual period (LMP) is often poor or inaccurate in settings with low rates of literacy and antenatal care. Access to ultrasonography is low and mothers frequently present for ANC late in pregnancy, when ultrasound dating is inaccurate. Thus improved assessment of gestational age before and/or after birth, and documentation of gestational age in medical records, is an urgent priority in all settings irrespective of resource availability, along with improved tracking of safety and non-fatal outcomes. Studies are needed to validate different and feasible methods of ascertaining gestational age compared to accurate gestational age dating (early ultrasonography) in LMIC. Furthermore, methods require validation in different regions and in settings with high rates of fetal growth restriction.

Thus, present capacity within most LMICs may only extend to crude coverage of ACS (e.g. all mothers who received 1 dose) and will not differentiate between those who received ACS before (true positives), or after (false positives) 34 weeks completed gestation. To capture such information, existing datasets from high or middle income countries may be analysed to facilitate the development and testing of a more refined indicator. Improved gestational age assessment and documentation is needed in all settings irrespective of resource availability, along with improved tracking of safety and non-fatal outcomes.

Observation of facility births in a number of countries would allow for testing and validation of a number of options for the denominator (Figure 5). The measurement improvement roadmap aims to assess whether using these denominators is feasible in routine HMIS, and the extent to which proposed options for testing yield useful programmatic tracking information.

As with many of the treatment intervention coverage indicators, the option of using all live births as a denominator will not give accurate population-representative treatment coverage in settings where reporting in HMIS is poor, such as settings with low facility births or a large private sector. In such contexts it may be worth considering estimated births within a facility catchment area as denominator, which is more challenging where populations are not well defined or birth cohorts are uncertain. A denominator that is not restricted to the population in need, will require definition of target coverage levels. For ACS this target benchmark could potentially be defined by the recent estimates of national preterm birth rate (<34 weeks), which was shown to vary from around 4% to 18% globally [41].

### Neonatal resuscitation

#### Technical definition of intervention

Basic neonatal resuscitation describes assessment and actions for every newborn at the time of birth, to assist in establishing breathing and circulation [42]; it should be practised on all non-macerated newborns not breathing spontaneously following immediate drying in accordance with current WHO guidelines [43]. Effective and safe resuscitation of these babies is highly time-sensitive and should be initiated within the first minute after birth. The actions include additional stimulation and positive pressure ventilation with bag and mask if clinically indicated following stimulation [44]. The intervention definition does not include advanced resuscitation measures such as intubation and/or medications.

#### Current coverage data availability

National coverage data are not currently available on neonatal resuscitation and the intervention lacks a standard measurable indicator. As with ACS, there are several known and suspected limitations of using household surveys to measure neonatal resuscitation coverage, including the likely inability of mothers to report accurately as they may not understand or know if their newborn was resuscitated at birth, and small numbers resulting in low statistical power [12,45].

#### Process indicator to measure now

Data on the availability of a functional newborn size bag and mask in the delivery area of a health facility offering maternity services may be utilised as a service readiness indicator for neonatal resuscitation, as these data are easy to document and already available now for many countries (see Figure 5) [16-18]. SPA and SARA capture the availability of at least one neonatal size bag and mask in the labour and delivery ward (SARA captures two sizes of masks) and neonatal resuscitation was added to the UN EmOC signal functions in 2009 with data collected as part of standard EmOC needs assessments supported by UNICEF. Since a neonatal-size bag and mask is on the UN essential commodities list, this equipment is also increasingly tracked in logistics management information systems (LMIS). This indicator has strong negative predictive value (a labour ward with no bag and mask cannot ensure adequate resuscitation when needed) and was recommended by the WHO consultation on quality of care [46]. However, the presence of resuscitation equipment does not equate to appropriate and timely use of the neonatal bag and mask, and not all newborns who do not breathe at birth require positive pressure ventilation. Many newborns may respond to stimulation alone, and there is evidence demonstrating that the provision of resuscitation training is associated with a reduction in bag and mask use [47]. Supplementary information regarding the presence of staff who have received newborn resuscitation training in the last two years is collected as part of the SARA and SPA surveys; however, these data may be difficult to compare depending on question framing [16,17].

#### What can we do to improve the data?

One of the major challenges in capturing precise neonatal resuscitation coverage is the identification and accurate measurement of a denominator that reliably captures babies requiring resuscitation to establish breathing after birth. As with other treatment indicators, accurate identification of the target population depends on correct diagnosis and classification of the individuals in need by health care providers. Accurate classification of babies needing resuscitation is challenging in all settings due to variable diagnostic skills and experience of individual providers [45,48]. Independent of provider competence, this would likely be difficult data to collect in routine systems; we can speculate that it is unlikely that any healthcare worker would record a case where a baby required resuscitation but did not receive it. As with ACS, the measurement improvement roadmap outlines the priority denominators for testing and the validation of observed compared with reported resuscitation practises. Appearance, Pulse, Grimace, Activity, Respiration (APGAR) scores were intended to assess the condition of the newborn after birth, but are not useful for measuring of resuscitation for monitoring purposes as they are not reported until 1 minute of life, after the time within which resuscitation should be initiated. In addition, APGAR scores may not be predictive of outcome unless the score is very low at 5 minutes, and in busy labour wards the scores are often recorded after the event, if at all.

There are further challenges associated with defining a numerator to accurately and feasibly track neonatal resuscitation coverage. An important principle in effective and safe neonatal resuscitation is careful assessment and stimulation of the newborn who does not start breathing spontaneously after routine drying, and only using bag and mask if needed in order to reduce inappropriate use of positive pressure ventilation [44,49,50]. However, bag and mask use may be easier to recall and validate than distinguishing stimulation actions, such as back rubbing, from routine drying and wrapping. A study in Sweden found that neonatal resuscitation documentation was inadequate for reliable evaluation [51]; documentation of resuscitation is unlikely to be more adequate in LMICs. Several countries such as Bangladesh, Nepal and Tanzania, propose testing collection of routine information on newborn resuscitation by action step. Further analysis of such efforts is likely to be useful.

Proposed testing includes comparison of health worker documentation of newborn resuscitation actions in facility records with observed or video recorded resuscitation care; some of this may be possible using existing videos from Nepal or birth records from Bangladesh. New work to observe births in health facilities across a number of countries would allow testing of the resuscitation denominator options (Figure 5) in line with the other treatment indicators, including various case definitions of babies who do not breathe at birth, or do not breathe after stimulation. A simpler denominator for resuscitation based on live births would require defined target levels. According to estimates (based on limited observational data) approximately 6-10% of newborns may require some assistance to begin breathing at birth [48,52].

### Kangaroo mother care

#### Technical definition of intervention

A method of caring for low birthweight newborns (mostly preterm) in direct and continuous skin-to-skin contact, in the kangaroo position, with their mother (or guardian), with support for early and exclusive breastmilk feeding. The current evidence to achieve mortality reductions supports KMC for clinically-stable newborns, weighing less than 2000 g, initiated in a facility [53]. WHO guidelines support that the infant is cared for in the kangaroo position for the equivalent number weeks it would have taken for the infant to reach full term (or as long as the baby will tolerate the position) accompanied with appropriate follow up after discharge [54].

#### Current coverage data availability

Limited data on KMC are available from facility-based surveys and HMIS for several countries, including Malawi, Dominican Republic, and El Salvador. Some middle-income countries, especially in Latin America, have detailed program data on KMC received, but there is no existing standardised coverage indicator definition. There may be differences between the level of facility in which KMC can be safely provided or initiated and the eligibility criteria for KMC, which creates difficulties in comparing data between settings. Measurement of KMC is not currently carried out by routine household survey platforms.

#### Process indicator to measure now

Given the immediate challenges for capturing coverage, a service-readiness indicator is proposed: the number of facilities in which a space is identified for KMC and where at least one staff member has received KMC training (SPA measures within the last 2 years) (see Figure 5). This measure is similar to that defined in a recent consultation by WHO on improving measurement on the quality of maternal, newborn and child health care in facilities [46] and is consistent with current SARA and SPA facility assessment tools [16,17].

#### What can we do to improve the data?

It is possible to measure the number of newborns initiated on facility based KMC in a number of settings through HMIS or hospital admission records (e.g. El Salvador, Dominican Republic, Malawi, Tanzania). However, measuring a denominator of <2000 g is challenging given that nearly half of all newborns globally are not weighed at birth. Where birthweight is recorded, there is a known tendency for digit preference and heaping, especially at 2500 g and 2000 g [55]. The denominator could be measured as a rate per 100 or per 1000 live births, avoiding the difficulties of including weight in the numerator and identifying babies in need for the denominator. However, this doesn't measure whether babies were truly eligible or benefitted from KMC. Since KMC is an intervention that benefits predominantly preterm infants, the proportion of live births that could benefit from KMC will vary between settings (4 to 18%); identical rates may correspond to a different unmet need for KMC [41].

Efforts to improve birthweight recording and gestational age assessment are integral to the scale-up and measurement of more precise indicators for KMC. Existing datasets from countries with established KMC programmes and accurate assessment of gestational age and birthweight should be used for testing the denominators and proposed numerators (Figure 5). Linked to the measurement improvement roadmap, developing and validating questions for household surveys is also important if the practice is widespread enough to ensure a sufficient sample size. Recent work in Colombia has shown that women can accurately and reliably recall KMC, even decades later [56].

To develop the service readiness indicator, both the WHO quality of care report and the KMC Acceleration Group propose a measure of "operational" KMC [46], although this would need further work to identify and test its specific components. The operational indicator could be based on available "tracers"; for example, SPA currently collects data on allocated KMC space, infant weighing scales, thermometer, and whether staff has received training. Other items (feeding cups, NG tubes, job aids) or improvements to the questions on training and space could be added where more detailed assessments are being carried out. In Colombia, a manual of minimum, desirable and optimal standards for KMC has been developed [57], which could be adapted for different settings.

### Treatment of neonatal infection

#### Technical definition of intervention

The provision of antibiotics to newborns admitted for inpatient care with possible serious bacterial infection (pSBI), in accordance with current WHO treatment guidelines [58,59] and diagnostic algorithms [60]. Case management can also be considered by levels of care: administration of oral antibiotics only, injectable antibiotics only, or full case management of neonatal infection (potentially second line antibiotic therapy, IV fluids, oxygen therapy, other supportive measures) [61]. Recent trials of Simplified Antibiotic Therapy show that, where referral is not possible, treatment with the simpler regimes by lower level workers is feasible [62].

#### Current coverage data availability

Most LMICs do not collect or aggregate the number of newborns treated for pSBI in HMIS. Household surveys, including DHS and MICS, do not collect data on newborns treated for pSBI because these would likely be unreliable (given recall issues measuring incidence of pneumonia in children under five years) [63]. This contrasts with HIC settings where HMIS data is routinely maintained with additional data points specific to monitoring antibiotic resistance.

#### Process indicator to measure now

Given challenges in measuring coverage of treatment of serious neonatal infection, a process indicator is proposed: the proportion of facilities in which gentamicin is available (at a suitable peripheral level) for treatment of serious neonatal infection [46]. This is collected by both the SPA, SARA facility assessment tools [16,17] and the WHO health action/access international database [39]. However, as with resuscitation, the presence of the antibiotic in the facility does not directly measure correct use of antibiotics to treat newborns for pSBI or guarantee that the antibiotic is available in paediatric doses [64].

#### What can we do to improve the data?

The number of newborns treated with at least one dose of injectable antibiotic at a facility is proposed for validation and feasibility testing against a number of denominator options, including total live births, the number of newborns presenting with illness, or the number of newborns diagnosed with pSBI (Figure 5). As treatment regimens may vary between settings, the measurement improvement roadmap aims to assess multiple options for a numerator and explore the validity, feasibility and utility of using HMIS to collect this data. For measurement of the dose of any antibiotic, more details would be required at program and/or facility level (rather than from the coverage indicator); notably, which antibiotic(s) were used and whether the course was completed. It will be necessary to determine appropriate use of antibiotics, as over treatment may increase anti-microbial drug resistance. Routine, national systems are required to track all injectable antibiotic doses given, and those not given, with associated clinical outcomes. A recent review found that within facility based audits, the availability of data on neonatal specific formulations (lower concentration gentamicin, procaine benzylpenicillin) was scarce [64] and therefore, more data is needed regarding the availability of neonatal formulations and specific requirements for administration to newborns. At first level facilities, testing of the new WHO module on "where referral is not possible" with new simplified antibiotic regimens [65] will be possible in five countries (Democratic Republic of Congo, Bangladesh, Pakistan, Ethiopia and Nigeria). Process and quality indicators should also be improved at the facility level, for example, gentamicin has a narrow therapeutic index and is associated with toxicity risks [58]; therefore, monitoring its safe administration at program or facility level is an important marker of quality care. Specific data on neonatal administration of medicines (formulations, concentrations) could also help monitor safety and quality of care in facilities. In addition, where direct patient observations are carried out (as with SPA for the treatment of suspected pneumonia), this could be extended to the treatment of serious neonatal infection in facilities to ensure health worker compliance with IMCI guidelines [59].

### Chlorhexidine cord cleansing

#### Technical definition of intervention

Chlorhexidine (CHX) cord cleansing is the routine application of topical chlorhexidine digluconate 7.1% (solution or gel, delivering 4%) to the cord stump within the first 24 hours of life. The WHO currently recommends this intervention in settings with an NMR >30:1000 or for homebirths [66,67].

#### Current coverage data availability

The recommended routine administration of chlorhexidine cord cleansing is a recent policy development [25]. Data are not collected by most HMIS or as part of standardised household survey tools. Both SPA and SARA track the availability of chlorhexidine used for general disinfection in their commodity checklists [16,17]. Monitoring use of 7.1% chlorhexidine for cord cleansing requires documentation of the presence of the specific concentration of chlorhexidine (7.1% formulation rather than any type of chlorhexidine product). Because of country-specific variations in policy for routine cord cleansing, documenting availability of 7.1% chlorhexidine in a health facility will only be of use in settings where programs that use chlorhexidine for umbilical cord cleansing exist.

#### Process indicator to measure now

Given the current challenges in measuring coverage, the inclusion of chlorhexidine 7.1% (solution or gel) within national essential drug lists for the purpose of cord cleansing has been identified as an interim process indicator (Figure 6). These data are collected by the RMNCH Trust (formerly UN Commodities Commission) and are reported by Countdown [20]. As with ACS, this indicator is distal and is not a measure of coverage; however, it is an important enabling condition, data are currently available, and it would facilitate tracking of policy changes in the coming years.

#### What can we do to improve the data?

Household surveys can be used to measure chlorhexidine coverage, as carried out in Nepal [68], *The number of newborns who had chlorhexidine applied to the cord stump within the first day of birth *can be evaluated against a denominator of live births in the survey population. DHS has incorporated an optional five question chlorhexidine module for countries with a national chlorhexidine for umbilical cord cleansing programme as part of its newborn module. In countries where chlorhexidine has been introduced at scale (e.g. Nepal, Bangladesh and Nigeria), the chlorhexidine technical working group is recommending adding a follow-up probe question specifically asking about chlorhexidine use.

Refinement of both the numerator and denominator with rigorous assessment of sensitivity and validity will be beneficial. Showing the respondent a picture of the locally marketed chlorhexidine during a household interview might assist with recall, improve validity and will be tested as part of the measurement improvement roadmap. Due to variations in national policy on use of chlorhexidine within facilities, further testing is required to assess the sensitivity and specificity of household survey questions on chlorhexidine cord cleansing following birth within a facility, where cord cleansing may have occurred away from the mother, or performed in her absence. Further validation will be undertaken to compare observed chlorhexidine use with reported practice. Depending on findings, longer-term efforts towards institutionalising chlorhexidine coverage questions within routine household survey platforms would be essential to achieve consistent coverage data.

## Discussion

The *Every Newborn *movement is committed to supporting countries to reach a target of ≤12 neonatal deaths and stillbirths per 1000 births by 2030, also closely linked to ending preventable maternal deaths [7]. The ENAP metrics process has highlighted major gaps and lack of tracking for newborn interventions at all levels of the health management information system. To date, insufficient technical work and investment has been dedicated to strengthening national data systems and to rigorous testing of coverage data. Both validation and feasibility testing using standard research protocols for rigorous testing are needed. The multistage ENAP metrics process identified 10 core indicators and a set of 10 additional indicators (Figure 2). Of the core ENAP indicators, five newborn-specific interventions are high impact and central to ENAP, yet coverage information is not collected through existing measurement platforms with comparable data. Our findings highlight the priority actions required to improve ENAP indicators, especially coverage, and detail the technical and research priorities that will enable countries to collect and use the data in health sector review processes (Figure 1); these findings are informing a roadmap to address measurement deficits by 2020.

### Measurement improvement roadmap

**Figure 7:**
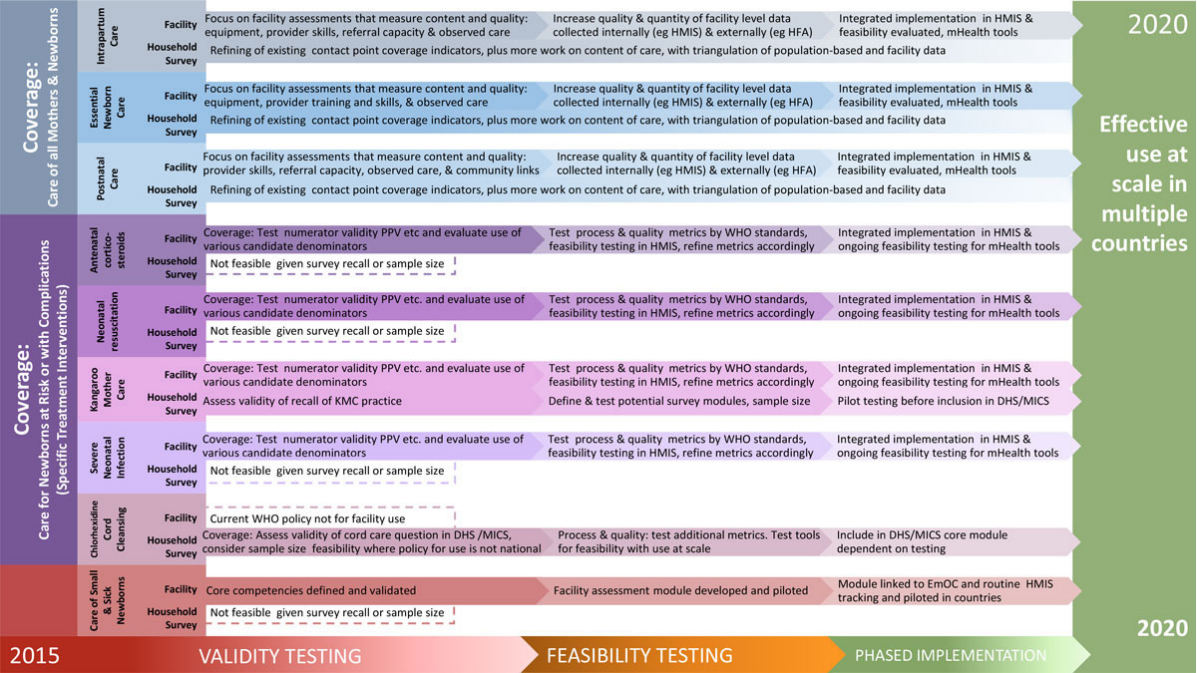
**Measurement improvement roadmap for coverage indicators (including care of small and sick newborns)**. DHS: Demographic and Health Survey, HFA: Health Facility Assessment, HMIS: Health Management Information System, MICS: Multiple Indicator Cluster Survey, PPV: positive predictive value, WHO: World Health Organization.

**Figure 8:**
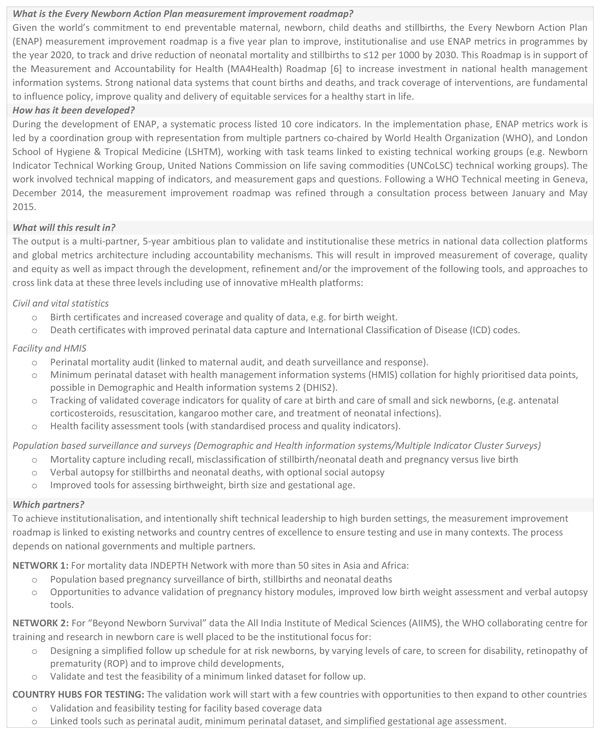
***Every Newborn *Action Plan (ENAP) Measurement Improvement Roadmap**. ENAP: Every Newborn Action Plan; HMIS: Health Management Information System; WHO: World Health Organization.

The ENAP measurement improvement roadmap aims to build on existing national and global metrics work, particularly linking to maternal health metrics, whilst identifying and addressing key measurement gaps for the focus around care at birth and care of small and sick newborns (Figure 7). Through this process the measurement improvement roadmap aims to intentionally transfer data collection, management and analysis skills at a country level (Figure 8).

#### Impact indicators

Impact indicators are fundamental to tracking progress for *Every Newborn*. Without impact data we cannot accurately measure progress towards goals to end preventable maternal and newborn deaths and stillbirths. Child mortality data have seen the most significant improvement progress over the last decade [69]. For example, the UN Inter-agency Group for Child Mortality Estimation report more than tripling input data, mostly through surveys.

ENAP milestones by 2020 include a number of tools to link facility-based minimum perinatal datasets with CRVS to increase birth/death registration [70] and birthweight capture, and in settings with a high proportion of home births, links to intermittent surveys or population surveillance may also be possible (Figure 1). Some countries are now implementing maternal death surveillance and response [71] and have begun to count maternal deaths in real time. A few countries are also incorporating perinatal death audits, which represents a key opportunity to expand use and quality of perinatal audit data [72]. A major focus is needed for inclusion of stillbirth rates in reporting and accountability mechanisms, and especially increasing data on intrapartum stillbirths. Further opportunities have been identified in increasing the coverage and quality of CRVS and verbal autopsy to improve cause of death estimates for maternal, neonatal and stillbirths [73,74]. Substantial work is required on the additional indicators measuring newborn morbidity, disability and child development, which are critical to validate and institutionalise particularly as countries scale up neonatal intensive care services (Figure 7).

Improving measurement of gestational age is essential given that prematurity is the leading cause of newborn deaths and deaths in children under five [75]. Preterm birth is also a major risk factor for deaths from infections and other morbidities [76]. Gestational age is an essential part of clinical targeting of interventions to reduce morbidity and mortality and can be measured both during pregnancy (using methods ranging from the dating of LMP to using more resource intensive ultrasound scans) to clinical assessments of the newborn. The skill sets needed for the measurement approaches that are currently available are different. Estimating gestational age using first trimester ultrasound and the date of last menstrual period is standard in most HIC, but these methods are not available for most women in LMIC. LMP recall is often poor or inaccurate in settings with low literacy. Universal access to ultrasonography is unlikely to be available to large numbers of women in LMIC in the shorter term, and/or mothers who present late in pregnancy, when ultrasound dating is inaccurate (+/- 3 weeks). Current work is looking at the potential for simplified tools for more accurate assessment of gestational age [77], including simplified clinical tools, and surrogate anthropometric measures that could be used by community health workers [78-80]. Validation of new methods in cohorts with early accurate ultrasonography dating is a critical need. Feasible and innovative approaches need to be validated in different regions, populations and settings, across which their performance may vary.

#### Coverage indicators

The next five years demands an ambitious and systematic process for data improvement (through effective partnership) to address the gaps in newborn coverage indicators. Shared goals across the MNH community will facilitate metrics testing and help institutionalise capacity for systems to collect and use these data (Figure 1). In the short term, desk-based testing and validation of indicator definitions using existing datasets (from LMIC) is required. Additionally, these indicators need to be field-tested in a range of settings. The research process for validation of indicators involves collecting empirical data through direct observations of care in a facility and directly comparing this data with both health worker reports and maternal recall of events. Relatively large numbers of direct observations may be needed to ensure sufficient sample power for estimating sensitivity and specificity of the indicators using appropriate statistical tests. Initial testing sites have been identified as part of the measurement improvement roadmap (Figure 8). Once finalised, testing protocols will be made available to facilitate wide-scale testing across many different settings to yield comparable results. Where indicator definitions already exist and are being collected at scale, there is potential to increase the quantity, quality and frequency of the data (Figure 4). Crosscutting work on increasing the availability, quality, and accuracy of birth weight and gestational age assessment (both in pregnancy and the neonatal period) is needed and will support the development of more precise indicators. It is anticipated that findings from these studies will inform refinements to the proposed indicators before institutionalisation into existing systems (Figure 7).

### Household surveys for tracking coverage

Household surveys remain the primary data collection method to estimate coverage of contacts with the health system. The Population Council is carrying out ongoing work to assess the validity of current indicators measuring skilled attendance at birth [81]. Such work provides invaluable evidence on the validity of maternal recall of interventions at the time of birth, with MICS using two year recall and DHS now using the last birth within two years for some maternal and newborn indicators (although collects data for a five year retrospective period). Even where recall achieves higher specificity (such as location of birth or Caesarean-section), their infrequent cycles (currently averaging 5 years) and high cost [82]) make population level surveys less sensitive for annual programme planning and timely decision-making [83]. Previous efforts to improve measurement of many interventions have focused predominantly on household surveys [12,26,84], including recent validation studies from the Improving Coverage Measurement Group. Many of the challenges of measuring the treatment of pneumonia in children through household surveys, especially in identifying the true population of children with pneumonia for the denominator [58], are also applicable to measuring coverage of treatment of neonatal infections and other specific treatment interventions.

**Figure 9:**
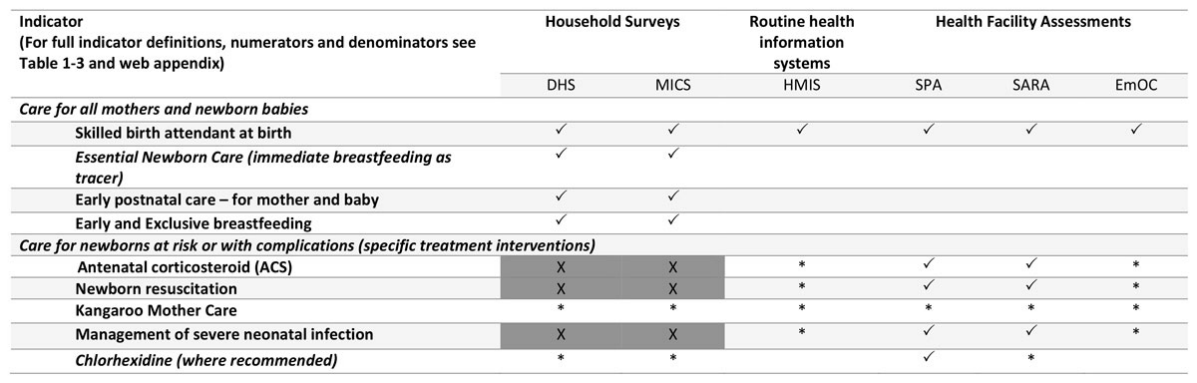
**Large scale data collection platforms for coverage and process indicators**. ✓ Already collected. * Feasible to collect. X=Not likely to be feasible to collect (due to recall of numerator, denominator identification challenges, sample size issues). DHS: Demographic and Health Surveys, MICS: Multiple Indicator Cluster Surveys, HMIS: health management information systems, SPA: Service performance assessments, SARA: Service Availability and Readiness Assessments, EmOC: Emergency Obstetric Care.

The sample size required to generate point estimates of coverage of newborn interventions with sufficient precision through household surveys is often too high; even more so when attempting to consider equity, and analyse by socioeconomic and demographic factors. For chlorhexidine cord cleansing in settings where policy is provision for all live births [25], data collection through a household survey such as DHS could be feasible. Other treatment indicators address subsets of newborns, and therefore, sample sizes and recall issues may make household surveys very challenging for coverage measurement. For measurement of treatment indicators, the results of the ENAP metrics process suggest a shift away from household surveys towards a focus on facility based data collection tools where these interventions can be more feasibly and accurately measured, and a range of denominators tested for use (Figure 9).

### Facility data for tracking coverage

For most of the treatment interventions, KMC, ACS, and currently most neonatal resuscitation and serious neonatal infection case management, policy recommendations are focused largely on facility-based initiation or administration. This has meant that preliminary task team work has focused predominately on facility platforms (with the exception of Chlorhexidine). Combined testing in a number of facilities of the range of treatment interventions would enable more efficient testing of a range of numerators and denominators for each intervention using the same datasets, and help to harmonise and align indicators with national and facility-level needs.

#### Where there is no denominator

Task teams found denominators the most technically challenging issue for measurement of intervention indicators and have identified a list of denominator options for testing wherever possible. Where detailed datasets are available with complete and accurate birthweight and gestational age data (for example in higher or middle income settings), these will be analysed to test and compare the simplified per 100 or per 1000 live births denominator to a more precise indicator option to ascertain correlation between risk and the more precise indicator, and sensitivity to change over time.

In view of contextual variation, such as varying preterm birth rates, or pSBI in different countries, there may be a need to define thresholds or upper and lower limits for indicator values. The proportion of C-section deliveries, for example, has been roughly benchmarked against a threshold of 5-15% in order to highlight where there is an unmet need (less than 5%) or to identify an excess number of C-sections (more than 15%) within a population [85-87]; this threshold is not without controversy. Learning from such processes is important to set realistic, useful ranges for countries to monitor whether interventions are reaching a sufficient number of newborns within safe limits.

#### Health management information systems

Work towards sustainable, real-time, locally owned and used systems underlines the need for strengthening national HMIS [83]. HMIS refers to health information collected and routinely reported from health facilities and districts (often from government or public sector facilities only) and are an ideal platform to influence as they are present in most settings, relatively inexpensive (compared with large scale representative household surveys) and largely driven by national decision makers. Electronic platforms are evolving to support data collection, management, analysis and report generation, linking to other systems including logistics management (rather than external agencies). The emphasis for strengthening HMIS needs to fall on improving the validity of HMIS indicators and increasing the use of this data for improving programme performance at the ground level. Many settings are now using District Health Information Systems 2 (DHIS 2)[88]. DHIS 2 software has a field-tested flexible data model with data entry forms for indicators and the ability to support data collection, management and analysis, including generating reports to monitor indicator trends over time and produce maps to visualise subnational variations for identification of inequities. There is potential for newborn treatment indicators (particularly KMC, ACS) to be included in HMIS/ LMIS, SPAs and other facility audits along with the supplies and equipment for ACS, neonatal resuscitation and pSBI treatment in settings where they do not already exist.

Before recommending inclusion of indicators into any national data collection system, indicators will need testing for validity and then for feasibility and usefulness, as per the steps of the measurement improvement plan (see Figure 1 and 7). Given the ongoing tension between demand for more information for decision making, versus the need to be parsimonious with the number of indicators to avoid overburdening frontline workers and information systems, prioritisation of the ENAP treatment indicators for inclusion in these systems should be country specific and consider relevance to national policy and health system needs. Overloading an HMIS system with data can limit its usefulness and negatively affect data quality. In addition to validity testing, consideration of national data needs, existing levels of facility, infrastructure, resources and technical capacity is essential before introducing new indicators into a national HMIS. Furthermore, data from HMIS may be more limited in settings where a large proportion of births take place in the community (e.g. Ethiopia), or where there is a large private sector (e.g. India).

### Input and process data for tracking content and quality of care

Given the challenges in measuring coverage for several of the treatment interventions, appropriate process indicators were identified that can be measured immediately. For the purpose of this discussion, "process" data refers to any measurement of the presence of specific elements needed to deliver an intervention, such as supportive policy, trained staff, commodities, documentation or infrastructure. Process data are not a replacement for coverage data, but ensure a standardised proxy can be used immediately. These data can be measured through a variety of platforms, including HMIS, routine audits and/or facility based supervision checklists. Additionally, periodic or intermittent health facility assessments, such as SPA [17] and service readiness assessments, such as SARA [16] and EmOC needs assessments [15] monitor process indicators. As many of the indicators (impact and coverage) measured through household surveys require relatively long periods of time to see significant change following policy adjustments, facility level programmatic data is essential for measurement of more proximate factors in the facility that are more amenable to change in the shorter term. Furthermore, facility surveys can provide external validation for self-reported data, such as those emerging from HMIS. Harmonisation of core modules for HFAs should include the priority ENAP process indicators to maximise their use and allow for comparison between surveys (Figure 9). However, the use of periodic health facility assessments is expensive and does not replace routine supervision or programme monitoring.

Some task teams proposed indicators regarding existence of supporting policy at national level as a key measure of process. For example, the task teams for both ACS and CHX proposed a measure of the number of countries with ACS or CHX respectively on the essential drug list since their addition is recent (2013) [37]; data are collected in the RMNCH Trust data system and reported in Countdown (Figures 5 and 6). Given that these interventions are at an earlier point in policy to programme change, these may be useful trackers for now but as programme implementation accelerates, the process indicator should be shifted to more proximal readiness indicators and coverage.

The ENAP measurement improvement roadmap, in partnership with other tracking data harmonisation efforts, aims to test both simple and composite readiness indicators for newborn interventions, considering the presence of essential commodities, trained staff, and space.

#### Care of small and sick babies

There is a major gap in the definition of standards for the care of small and sick newborns; the provision of quality inpatient care for small and sick babies could have a significant impact on neonatal deaths [9]. The UN EmOC indicators are based on process indicators referred to as "signal functions" for basic and comprehensive emergency obstetric care [18]; currently only one signal function specifically relates to newborn care, but does not fully represent all interventions needed for emergency newborn care. New research supports the addition of signal functions specific to newborn care and strongly recommends that these indicators should be updated [89]. Specific challenges and details on the levels of care are explained in greater detail elsewhere in the series [90] and ENAP recommends an ongoing process with the UN to define indicators for newborn care intervention packages by levels of care.

As a milestone from ENAP linked to EPMM, addressing quality of care at birth is critical; the Every Mother, Every Newborn (EMEN) Initiative is part of this process as discussed in paper 1 of this supplement [91].

### Challenges and opportunities going forward

#### Integrating maternal health and broader roadmaps for improving metrics

It is essential to unite maternal and neonatal health communities towards a common metrics agenda with a convergence of global efforts to end preventable mortality and coordinated support to countries to assess progress meeting targets set within the SDGs, ENAP and the ending preventable maternal mortality movement (EPMM). These functions are the remit of the WHO, other UN agencies and academic partners, and can be aligned through the creation of an over-arching MNH reference group. This remit will also aim to link existing work and relevant convening groups, including those working on wider metrics systems change.

#### Intentional development of leadership to assess, improve and use data

In order to institutionalise the proposed metrics, there is a need to build leadership skills to assess and use data in high burden settings (Figure 8). These include INDEPTH Network's Maternal and Newborn Working Group, which aims to improve population-based metrics, especially pregnancy tracking, mortality, cause of death and social autopsy, birthweight and gestational age. INDEPTH is a network of currently 52 health and demographic surveillance sites (HDSS) in twenty countries where a total population of 3.8 million people are tracked each year. The Maternal and Newborn Working Group is building the capacity of member sites to use standardised tools and to make data regularly available to the public. The All India Institute of Medical Sciences/WHO Collaborating Centre for Newborn Care is well placed to develop a simplified database for follow up of at risk neonates, track and minimise disability outcomes and maximise child development, especially preterm, for example preventing blindness from retinopathy of prematurity [92,93]. ENAP is identifying provisional country hubs for testing of proposed indicator numerators and denominators initially linked to focus countries for EMEN.

## Conclusions

**Figure 10:**
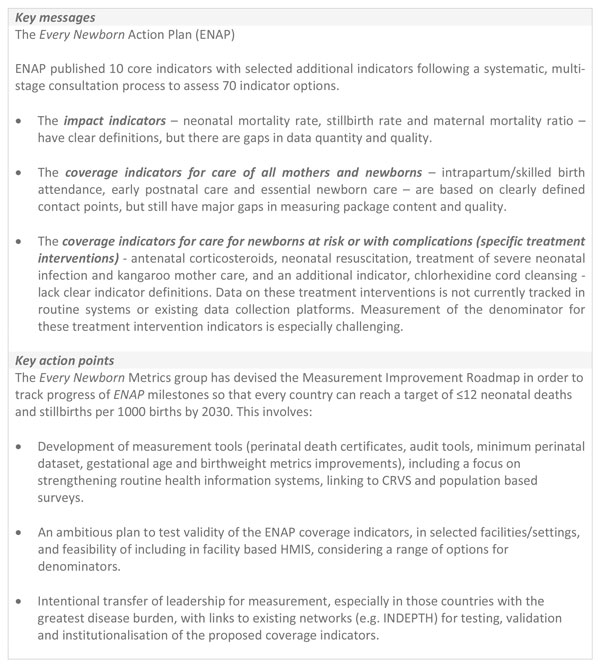
**Key messages and action points**. ENAP: Every Newborn Action Plan; HMIS: health management information systems.

Major gaps have been identified in the measurement of core ENAP indicators to track the progress towards targets to end preventable deaths for women, stillbirths, newborns and children; key messages and action points are summarised in Figure 10. The quality and quantity of impact data must be improved, but coverage indicators need the most urgent work. Content and quality of care is the current priority for the three contact point indicators. For the treatment indicators, preliminary work to identify measurable denominators is required in preparation for the quality improvement agenda. The findings of this work underline the need for increasing prioritisation for strengthening and improving routine facility based data, CRVS and national HMIS. This paper has laid out a systematic, yet ambitious testing agenda - the ENAP Measurement Improvement Roadmap - to move towards use of these indicators at scale, which must be combined with an intentional transfer of technical leadership, especially to countries with the greatest disease burden. The strengthening of institutional capability to collect, analyse and convert data into action is essential. By 2020, the aim is to institutionalise the proposed metrics at scale across all countries. A roadmap that focuses on counting births, deaths and improves tracking of coverage and equity is central to support countries to build a strong national data system that can be used to inform policy and focus investment and resources towards quality service delivery for every newborn to have the chance of a healthy start in life [6].

## List of abbreviations

ANC: antenatal care; ACS: Antenatal Corticosteroids; BFI: Baby Friendly Initiative; CD: Countdown to 2015; COIA: Commission on Information and Accountability; CHX: Chlorhexidine; CRVS: Civil Registration and Vital Statistics; DHS: Demographic and Health Survey; DHIS2: District Health Information Software 2; EMEN: Every Mother Every Newborn; EmOC: Emergency Obstetric Care; ENAP: Every Newborn Action Plan; HBB: Helping Babies Breathe; HFA: Health Facility Assessment; HIC: high income countries; HMIS: Health Management Information System; ICD: International Classification of Disease; KMC: Kangaroo Mother Care; LMIC: Low and middle income countries; LMIS: logistics management information system; LMP: last menstrual period; LSHTM: London School of Hygiene and Tropical Medicine; MCHIP: Maternal and child health integrated program; MDSR: Maternal Death Surveillance and Response; MICS: Multiple Indicator Cluster Survey; MMR: maternal mortality ratio; MNCH: maternal newborn and child health; NMR: neonatal mortality rate; PNC: postnatal care; pSBI: potential severe bacterial infection; QoC: quality of care; R-HFA: Rapid Health Facility Assessments; ROP: Retinopathy of Prematurity; RMNCH: Reproductive, Maternal, Newborn and Child Health; SARA: Service Availability and Readiness Assessments; SBA: Skilled Birth Attendant; SBR: Stillbirth rate; SGA: small for gestational age; SoWC: State of the World's Children; SPA: Service Provision Assessments; UN: United Nations; UNCoLSC: United Nations Commission on Life Saving Commodities; UNICEF: United Nations International Children's Emergency Fund; UN-IGME: United Nations inter agency group for child mortality estimation; UNFPA: United nations Population Fund; USAID: United States Agency for International Development; VR: vital registration; WHO: World Health Organization.

## Competing interests

All authors declare they have no competing interests. The content of this article is the view and responsibility of the authors alone and does not necessarily reflect the policy or views of any of the organisations listed, including: the World Health Organization, USAID or the United States Government.

## Authors' contributions

JEL and SGM conceptualised the paper coordinated the drafts with HR-F and MM. The ENAP Metrics coordination group ((JEL, MM, AA, SF. JG, ACM, LMEV) all reviewed and input starting from early drafts. The Coverage Task teams for antenatal corticosteroids (ACS), kangaroo mother care (KMC), newborn resuscitation, severe neonatal infection case management, and chlorhexidine (CHX) cord cleansing all worked on their indicator and specific sections. All authors reviewed drafts and approved the final manuscript.

## Supplementary Material

Additional file 1 Listing of relevant indicators according to level of the impact framework (from impact down to inputs). Click here for file

## Declarations

Publication costs for this supplement was funded by the Bill and Melinda Gates Foundation through a grant to US Fund for UNICEF (Grant ID: OPP1094117), and support from Save the Children's Saving Newborn Lives Programme. Additional funding for the bottleneck analysis was received from USAID (Grant ID: GHA-G-00-07-00007) through UNICEF. CIFF funded the development of the ENAP measurement improvement plan.

This article has been published as part of *BMC Pregnancy and Childbirth *Volume 15 Supplement 2, 2015: Every Woman, Every Newborn. The full contents of the supplement are available online at http://www.biomedcentral.com/bmcpregnancychildbirth/supplements/15/S2.
